# Luminescence of thermally altered human skeletal remains

**DOI:** 10.1007/s00414-017-1546-1

**Published:** 2017-02-23

**Authors:** Tristan Krap, Kevin Nota, Leah S. Wilk, Franklin R.W. van de Goot, Jan M. Ruijter, Wilma Duijst, Roelof-Jan Oostra

**Affiliations:** 1 Department of Anatomy, Embryology and Physiology, Academic Medical Centre, University of Amsterdam, Meibergdreef 15, 1105 AZ Amsterdam, The Netherlands; 2 Department of Life Sciences and Technology–Biotechnology–Forensic Science, Van Hall Larenstein, University of Applied Sciences, Leeuwarden, The Netherlands; 3 Ars Cogniscendi Centre for Legal and Forensic medicine, Wezep, The Netherlands; 4 Department of Biomedical Engineering and Physics, Academic Medical Centre, Amsterdam, The Netherlands; 5 Forensic Technical Solutions B.V, Amsterdam, The Netherlands; 6 Centre for Forensic Pathology, Baarn, The Netherlands; 7 University of Maastricht, Maastricht, The Netherlands

**Keywords:** Luminescence, Bone, Heat, Cremation, Forensic anthropology

## Abstract

Literature on luminescent properties of thermally altered human remains is scarce and contradictory. Therefore, the luminescence of heated bone was systemically reinvestigated. A heating experiment was conducted on fresh human bone, in two different media, and cremated human remains were recovered from a modern crematory. Luminescence was excited with light sources within the range of 350 to 560 nm. The excitation light was filtered out by using different long pass filters, and the luminescence was analysed by means of a scoring method. The results show that temperature, duration and surrounding medium determine the observed emission intensity and bandwidth. It is concluded that the luminescent characteristic of bone can be useful for identifying thermally altered human remains in a difficult context as well as yield information on the perimortem and postmortem events.

**Electronic supplementary material:**

The online version of this article (doi:10.1007/s00414-017-1546-1) contains supplementary material, which is available to authorized users.

## Introduction

Recovery of human remains is of great importance in many contexts, such as accidents or crime scenes, as they aid the reconstruction of perimortem events as well as the identification process. Furthermore, there is an ethical obligation to recover as many of the remains as possible. The recovery of human remains from a scene involving thermal destruction can be difficult because, in most cases, the fragmentary human remains blend in with the structural or contextual debris. Hence, it is expected that not all remains will be recovered, which can have a negative impact on the interpretation of both the (crime) scene and the evidence, as well as on the identification of the deceased.

Bone goes through four gross stages when exposed to thermal stress. First, dehydration occurs (ranging from ±105 to ±600 °C), followed by decomposition of the organic matrix by pyrolysis and combustion (ranging from ±500 to ±800 °C). The third phase is characterized by inversion due to loss of carbonate resulting in calcination of the inorganic matrix with calcium oxide (CaO) and calcium hydroxyapatite (CHA) as solid end products. Additionally, a chemical conversion from CHA to β-tricalcium phosphate (β-TCP) is suggested to occur (from ±650 °C and higher). Finally, the inorganic matrix recrystallizes (from ±1600 °C and higher) [1]. These stages are associated with generally observed changes in bone colour, from ivory, yellow-white (fresh bone) to brown-black (carbonized bone) and bluish grey-white (calcined bone), minor variations set aside [2–7]. As indicated, the temperature ranges related to these stages overlap. Moreover, besides temperature, also duration of exposure, oxygen availability and distance to the heat source contribute to the change of colour. Finally, the duration of tissue shielding, which is related to more than one of these major variables, plays a role in the discolouration process [6–8].

Fluorescence is currently employed as a tool to detect various biological traces [9]. To improve the recovery yield of osseous material from difficult contexts, alternate light sources (ALS) have been suggested [10, 11]. An ALS emits light of a specific centre wavelength and limited spectral bandwidth. By stimulating molecules with a specific spectral bandwidth, the molecules can reach higher energy states. Rapidly thereafter, the molecules will lose some of the gained energy to their surroundings and subsequently return to their ground state, by emitting light. This emitted light is called (photo) luminescence. Luminescence can be divided into two pathways, namely fluorescence and phosphorescence [12]. The difference between fluorescence and phosphorescence lies in the decay time associated with the excited state multiplicity [13]. Luminescence can be distinguished if the excitation light is filtered out by a long pass filter [14]. It should be noted that fluorescence and luminescence are being used interchangeably as synonyms in some of the cited literature. Differentiating between the two independent pathways is impossible by means of an ALS.

Bachman et al. found that fresh whole bone exhibited a major emission peak at 440 nm when excited with 365 nm (ultraviolet light (UV light)) and two minor peaks at 590 and 640 nm, respectively. Both the inorganic as well as the organic components of bone (hydroxyapatite and type 1 collagen) were determined to be fluorescent [15]. Later, Craig et al. showed that the excitation spectrum of bone extends beyond the UV and far into the visible light spectrum [11]. Warren et al. suggested that all cremated human remains should be investigated with UV light and stated that cremated human remains of the same “age” and cremated in the same furnace fluoresce similarly [16]. On the contrary, Mavin found that cremated skeletal remains did not fluoresce under any light source in combination with any filter but did observe a dark purple colour when cremated bone was illuminated with a wavelength of 450 nm and viewed through a yellow long pass filter [17]. Some of the contradictory findings of Mavin and Warren et al. have been cited in recent literature [18–20]. Harbeck et al. showed that animal bone heated at both 200 and 400 °C fluoresced at UV excitation, exhibiting a brown colour, while samples heated at 300 °C did not; samples heated at 500 °C and higher appeared to be violet-brown to violet. Harbeck et al. also investigated cremated human remains from a modern crematory with UV and observed a bright violet fluorescence [21]. These opposing findings highlight, as stressed by Warren et al., that the origin of the fluorescent characteristics of heated bone is still unknown [20].

Hypothetically, if the inorganic component of bone also fluoresces by itself, it is to be expected that heated bone, exposed to a relative high temperature, will still fluoresce as long as the inorganic component is not changed chemically. However, to our knowledge, the available literature contains no empirical tests of this hypothesis. Gallant already showed the possibility of visualizing remains in a difficult context, mainly composed of fire debris, by inducing fluorescence with UV and a yttrium aluminium garnet (YAG) laser [22]. The question whether cremated human remains can be visualized by using a conventional ALS should be re-addressed given the contradictory findings of previous studies mentioned in this manuscript. Moreover, differences in intensity of luminescence could be useful for improved visualization of the heat line and heat-altered border, the area that was exposed to thermal stress but also protected by the retracting soft tissues, as was suggested by Schiers et al. [8, 23]. However, so far, no explanation has been provided for interpreting the observed differences in intensity and whether the presence of soft tissue has an effect on the thermal degradation of the bone matrix. Lastly, it is unknown if the emission bandwidth and centre wavelength change with exposure to thermal stress and which excitation wavelengths provide the best overall results. Consequently, there is a need for a systematic investigation of the luminescent properties of thermally altered remains.

To systematically investigate the luminescent properties of thermally altered human bone, a heating experiment was carried out on human long bones of varying sizes. The experiment was conducted in plain air and with subcutaneous fat as surrounding matrix. The experimentally heated bones and industrially cremated remains of four deceased were analysed by means of 11 ALS–long pass filter combinations, and the intensity was scored based on a scoring index.

## Materials and methodology

### Sample preparation and heating experiment

Skeletal material was extracted from unembalmed human cadavers. The left and right radii, ulnae and humeri from two cadavers were used, one male (age at death 66 years) and one female (age at death 75 years). The cadaveric material was obtained through the body donation program of the Department of Anatomy, Embryology and Physiology of the Academic Medical Centre, Amsterdam, the Netherlands. The bones were manually defleshed and stored between 4 and 7 °C. Thin transverse cross sections, of approximately 4 mm, were sawn with a bone saw from the radial and ulnar diaphyses and from parts of the humeral diaphysis, until the epiphyses were reached or the required number of samples was obtained. The remaining diaphyses of the humeri were divided into sections of approximately 40 mm thick. The bone was kept wet during sawing to prevent unwanted heating due to friction of the saw.

Thermal stress was applied for varying durations in a preheated muffle oven (with an accuracy of ±2 °C) in porcelain cups, up to a temperature of 1100 °C with increment steps between 20 and 100 °C. Two surrounding media were used, air and porcine subcutaneous fat (*Sus scrofa domesticus*). The latter was chosen to mimic the presence of soft tissue. Heating in adipose tissue was limited to a temperature of 450 °C because of rapid autoignition. The thin transverse cross sections were heated in air to a maximum temperature of 900 °C, since this covers the temperatures generally reached during a house fire [24]. The diaphyseal thick sections and epiphyses were heated up to 1100 °C, to enable a comparison with a modern crematory. The samples were heated and subsequently left to cool down to room temperature; details concerning the temperature, duration, medium and sample size are given in Online Resource 1 section A. During the entire process, the samples were handled with tweezers, and nitrile gloves were worn, to prevent contamination.

### Samples collected from a modern crematory

Four unembalmed, undefleshed and unaltered (prior to cremation) human cadavers, which were donated to science but unsuited for preservation, were recovered after a modern cremation. The sample population consisted of two males (age at death 77 and 81 years) and two females (age at death 77 and 83 years). Three of the four cadavers were kept refrigerated between 4 and 7 °C before cremation, and one cadaver was kept frozen and thawed prior to cremation (male, age at death 77 years). The postmortem interval prior to cremation did not exceed 2 days for the refrigerated cadavers and was 78 days for the frozen cadaver. The remains were cremated at a temperature of ±1000 °C for a duration of 2.5 h and salvaged prior to pulverization.

The cremated remains were handled with nitrile gloves. The salvaged material was sieved, and metals and other non-osseous materials were removed. The cremated remains were then categorized as cranial bones, teeth, vertebrae, ribs, irregular bones, epiphyseal ends and diaphyseal fragments.

### Visualization and imaging

The cortical surface of the thin transverse cross sections, the cortical, periosteal and articular surface of the diaphyseal ends and thick diaphyseal sections, and the remains collected from the modern crematory were illuminated with an ALS to induce luminescence. In total, five types of ALS were used: 350 to 380 nm (UV, peak at 365 nm), 400 to 430 nm (violet, peak at 410 nm), 420 to 470 nm (blue, peak at 445 nm), 445 to 510 nm (blue/green, peak at 475 nm) and 480 to 560 nm (green, peak at 520 nm) [25].

The samples were placed on a visually non-luminescent and strongly visible light-absorbing surface. The following long pass filter goggles were used to filter out the excitation light (1% transmission): 435 nm (pale yellow), 476 nm (yellow), 529 nm (orange) and 571 nm (orange). All combinations of ALS–long pass filter, higher than the excitation bandwidth of the ALS, were used in the experiment. A Nikon D700 with a 35-mm AF-D f2.8 lens was used for photographic documentation, in conjunction with long pass lens filters from Schott (1% transmission): GG455 435 ± 6 nm (pale yellow), GG496 476 ± 6 nm (yellow), OG550 529 ± 6 nm (orange) and OG590 571 ± 6 nm (orange). Digital images were taken in raw image format and postprocessed in Adobe Lightroom CC® (2015, Inc., San Jose, CA) for Mac. Contrast was enhanced by setting the levels appropriate to the image; the background surface was adjusted to black by manual selection in the majority of the images. No changes were made to the white balance, nor was the colour of the image enhanced.

### Excitation and luminescence interference

Spectroscopic measurements were performed to determine the actual spectral bandwidth of the ALS. This served the purpose of determining whether any illumination light would pass through the used filters and add to the observed luminescence. Measurements were recorded using a spectrograph (USB4000 from Ocean Optics, Duiven, NL), a standard multi-mode fibre (FT400EMT-M28L01 from Thorlabs, NJ, USA) and different long pass filters (400 LP 232, 450 LP 9604, FEL0500 and FEL0600 from Thorlabs, NJ, USA). The spectral output of the ALS exceeded the respective nominal cut-off wavelengths provided by the manufacturer. The five spectra are included in Online Resource 1 section B.

**Fig. 1:**
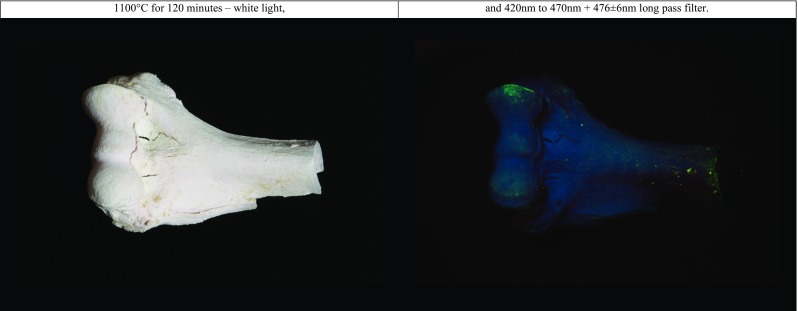
Observed homogenous illumination, observable bluish colour when excited with 420–470 nm and photographically recorded through a 476 nm long pass filter

In order to visually observe ALS output at wavelengths exceeding the spectral bandwidth specified by the manufacturer, and thus potential false positive luminescent observations, a mirror (PF10-03-P01 from Thorlabs) was used to inspect the reflectance. Several ALS–long pass filter combinations led to an observed reflection in the mirror. UV light reflected purple, although this was not observed in any of the photographs or when the sample was observed through the prism of the mirror reflex camera. A purple reflection was also observed when using the purple ALS with the pale yellow long pass filter. The blue ALS (420 to 470 nm) reflected blue-green in the mirror when observed through a yellow long pass filter (476 nm), as can be seen in Fig. 1. The blue-green ALS reflected green in the mirror, and the green ALS reflected yellow when observed through an orange-2 filter. The reflectance was relatively low in intensity, best described as a homogenous illumination, and disappeared when using the subsequent long pass filter.

### Scoring and statistical analysis

**Fig. 2:**
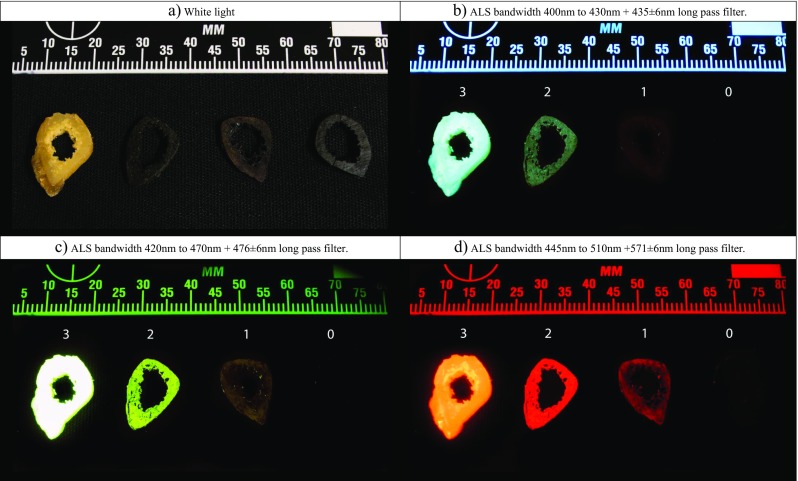
Four radial transverse cross sections under white light (**a**), 400 to 430 + 435 ± 6 nm filter (**b**), 420 to 470 + 476 ± 6 nm filter (**c**) and 445 to 510 + 571 ± 6 nm filter (**d**). Unheated (score 3)/400 °C for 20 min in adipose tissue (score 2)/300 °C for 30 min in air (score 1)/350 °C for 30 min in air (score 0)

To evaluate the effect of the thermal stress on the luminescent property of the bone, the luminescence was scored in a similar fashion as Ramsthaler et al.: present [strong] (4), present (3), present [weak] (2) or absent (1) [26]. Present [strong] was scored when the sample luminesced as intense as a fresh sample; present was scored when luminescence was evident but not as intense as a fresh sample; present [weak] was scored when only a slight amount of luminescence was observed, and absent was scored when no luminescence was observed, see Fig. 2 for a series of samples corresponding with the scoring index. This ordinal scoring index was used for each specific ALS–long pass filter combination. The previously mentioned reflectance for specific ALS–long pass filter combinations was discarded as false positive and thus not scored as luminescence. Two observers (TK and KN) scored a total of 260 samples, all in duplicate, with 11 ALS–long pass filter combinations.

#### Intraobserver and interobserver agreement

The samples were scored at two different moments by two observers in randomized order, without prior knowledge on the experimental treatments. The first and second scores by the observers were statistically compared by means of a kappa test to determine the intraobserver agreement. A kappa test was conducted on the four possible pairings of the duplicate scores of each of the observers to determine and interobserver agreement [27]. The kappa agreement scores were interpreted according to the suggested levels of agreement from McHugh [28]. Both observers achieved an almost perfect agreement for the kappa analysis on the first versus the second score; the κ values for this intraobserver agreement were κ 0.961 (*p* < 0.001) and κ 0.949 (*p* < 0.001), respectively. The kappa analysis of agreement between the observers ranged between κ 0.870 and κ 0.892 (*p* < 0.001), implying an almost perfect agreement between the two observers. Details on the kappa analysis are given in Online Resource 1 section C. Further statistical analysis was, therefore, performed on the mean of the two observations of both observers, for 11 ALS–long pass filter combinations for 260 samples.

#### Statistical analysis of temperature-dependent and duration-dependent changes of luminescence of bone in different media

Statistical analyses were performed in Microsoft® Excel for Mac 2016 and SPSS statistics for Mac. The overall mean score (with 2σ) was calculated and plotted for the temperature groups of the transverse cross sections heated in air and adipose tissue for 10, 20 and 30 min and of the diaphyseal thick sections and epiphyses heated in air at various durations.

The intensity scores of both the transverse cross sections heated in air and adipose tissue were compared with the Mann–Whitney *U* test to determine the significance of the difference between the different media. In order to determine the most efficient ALS–long pass filter combination, the various ALS–long pass filter combinations were compared with a Kruskal–Wallis H test; if a significant difference was found, a multiple comparison of groups, based on the mean rank, was performed to determine which combinations differed from each other. For all tests, statistical significance was accepted at *p* < 0.05.

## Results

### Luminescence of thermally altered thin transverse cross sections heated in air

**Fig. 3:**
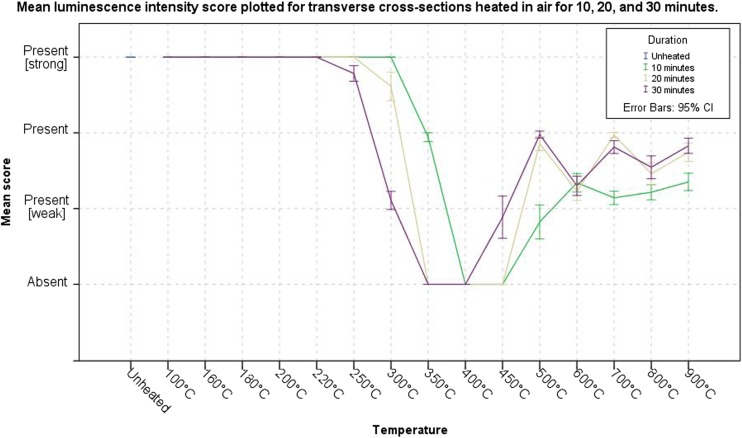
Graph of the obtained overall mean scores of the observed intensity of luminescence for the increasing temperature groups heated in air for 10, 20 and 30 min

**Fig. 4:**
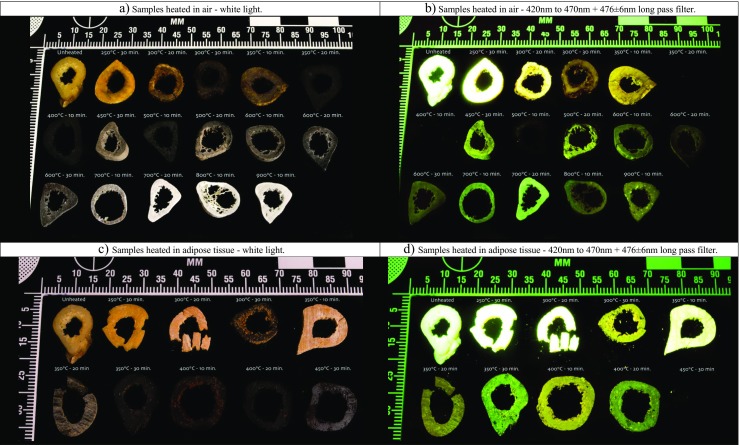
Variety of transverse cross sections, heated to different temperatures and durations in medium air (**a**, **b**) and adipose tissue (**c**, **d**)

The unheated transverse cross sections luminesced strongly when illuminated with any of the five ALS and observed through the long pass filters. This broad emission spectrum was observed for all samples, without a reduction in intensity, heated up to 250 °C. The first change in the intensity of the luminescence was observed at 250 °C after 30 min, followed by 300 °C after 20 min and 350 °C for 10 min. A prolonged duration at 300 °C, from 20 to 30 min, led to a decrease in intensity. The samples heated to temperatures in the range of 350 °C for 20 and 30 min and 400 °C for 10 and 20 min did not exhibit any luminescence. The luminescence reappeared at 450 °C after 30 min, and the samples heated to 500 °C for 10 min showed a similar reappearance of luminescence. Samples heated for 20 and 30 min at 500 °C exhibited a higher intensity than samples heated for 10 min at that temperature. Exposure duration had no effect on the intensity of the luminescence at 600 °C, while at temperatures from 700 °C and higher, the prolonged duration did result in a higher luminescence intensity. In general, for temperatures below 400 °C, a longer exposure duration led to a lower intensity, and at temperatures higher than 400 °C, a longer duration led to a higher intensity (Fig. 3). Figure 4a, b illustrates the described reoccurrence of luminescence.

### Luminescence of thermally altered thin transverse cross sections heated in adipose tissue

**Fig. 5:**
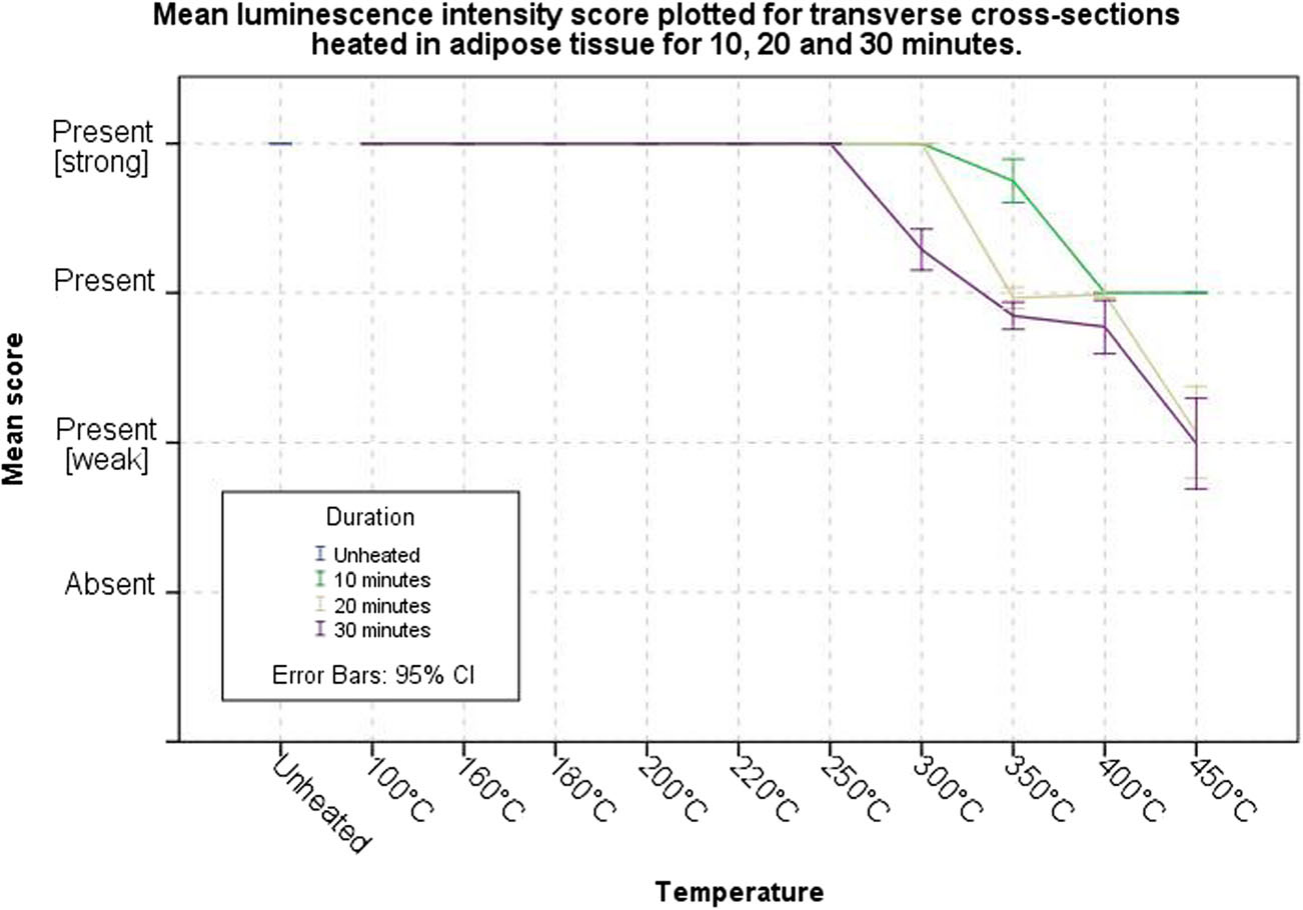
Graph of the obtained overall mean scores of the observed intensity of luminescence for the increasing temperature groups heated in adipose tissue for 10, 20 and 30 min

The luminescence of samples heated in adipose tissue is similar to an unheated sample up to a temperature of 300 °C for 20 min; after 30 min at that temperature, a lower intensity was observed. The intensity continued to decrease with increasing temperature and duration, similar to the samples heated in air. The samples heated to a temperature of 450 °C for 30 min obtained the largest standard deviation for the intensity score, ±0.5 (2σ) (Fig. 5). Figure 4c, d shows the luminescence of the samples heated in adipose.

The samples heated in adipose tissue luminesced stronger than the samples heated in air, up to a temperature of 400 °C (Figs. 3 and 5). The Mann–Whitney *U* test showed that this difference was significant between the two media for all temperature–duration groups, except for 450 °C and a duration of 30 min (Table 1).

**Table 1:** Results of the Mann–Whitney *U* test comparing the intensity of luminescence of transverse cross sections heated in air versus the transverse cross sections heated in adipose tissue

Group comparison heated in air × adipose tissue	Mann–Whitney *U*	Asymp. sig.
250 °C (30 min)	638.00	0.000
300 °C (20 min)	110.0	0.000
300 °C (30 min)	50.5	0.000
350 °C (10 min)	17.0	0.000
350 °C (20 min)	0.0	0.000
350 °C (30 min)	0.0	0.000
400 °C (10 min)	0.0	0.000
400 °C (20 min)	0.0	0.000
400 °C (30 min)	0.0	0.000
450 °C (10 min)	0.0	0.000
450 °C (20 min)	44.0	0.000
450 °C (30 min)	838.5	0.238

### Luminescence of thermally altered thick diaphyseal sections and epiphyses heated in air

**Fig. 6:**
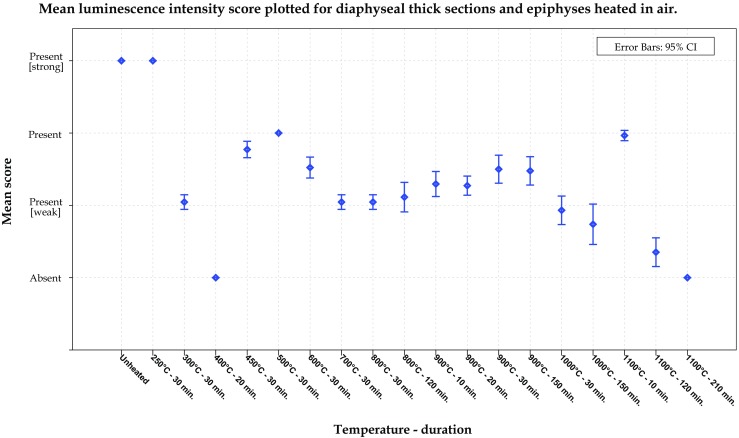
Graph of the overall mean scores for the observed intensity of luminescence obtained for the temperature–duration groups of the diaphyseal thick sections and epiphyses heated in air. The mean score is based on the observations by two observers for 11 ALS–long pass filter combinations

**Fig. 7:**
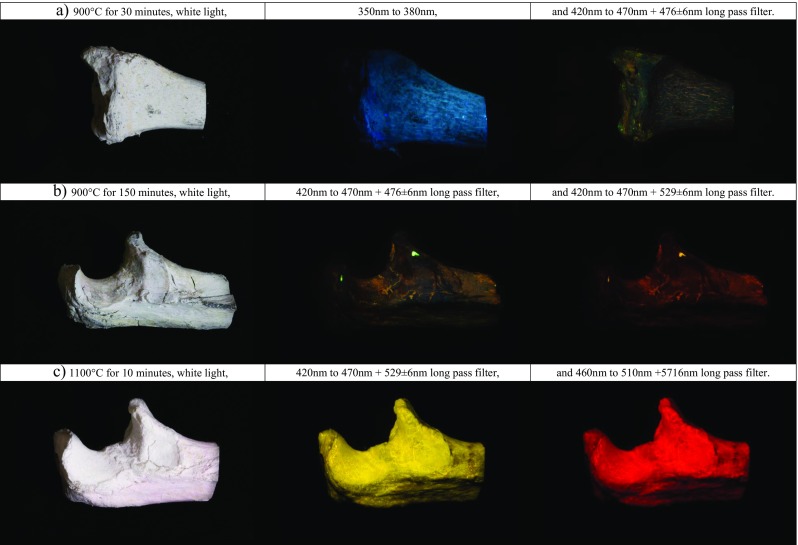
Epiphyseal ends heated to **a** 900 °C for 30 min, **b** 900 °C for 150 min and **c** 1100 °C for 10 min

The diaphyseal thick sections and epiphyses exhibited a similar trend in temperature-related changes when compared with the thin transverse cross sections up to a temperature of 900 °C. The first change in intensity of luminescence was observable at a temperature of 300 °C after 30 min. Samples heated to 400 °C for 20 min exhibited no luminescence anymore, after which a reoccurrence of luminescence was observed at 450 °C after 30 min. Samples heated in the range of 450 to 600 °C luminesced stronger than samples heated in the range of 700 to 1000 °C. The samples exposed to 1100 °C for 10 min luminesced stronger than the adjacent temperature groups; after 120 min, the samples still luminesced weakly, and after 210 min, no luminescence was observed (Fig. 6). The samples heated to 800 °C and higher displayed a different colour of luminescence, shifted from greenish to orange-red when illuminated with a bandwidth of 420 to 476 nm and observed through a 476 ± 6 nm long pass filter. Figure 7 shows the observed shift in emission bandwidth and a sample heated to 1100 °C for 10 min.

### Comparison of the effectiveness of different ALS–long pass filter combination on thermally altered bone samples within a specific [temperature–duration] range, heated in air

The temperature groups from 220 °C and higher, including all durations, both the transverse cross sections and the diaphyseal thick sections and epiphyses were combined to reach the required number of observations per group for subsequent statistical analysis.

The analysis with the Kruskal–Wallis test showed a statistical significant difference in observed intensity within 4 of the 13 groups within the range of 250 to 1100 °C, at 250, 600, 900 and 1000 °C. At 250 °C, the UV and violet ALS yielded a lower intensity score than the other combinations. The mean ranks of each ALS–long pass filter combination for 600, 900 and 1000 °C groups showed that the blue ALS with a yellow long pass filter yielded a higher observed intensity. At 1000 °C, the blue-green ALS with orange filter obtained a relative high observed intensity, whereas at 900 and 1000 °C, the UV light also yielded a relative high observed intensity. Table 2 shows the results of the Kruskal–Wallis test, and Table 3 gives an overview of the mean ranks for the groups that showed a significant difference.

**Table 2:** Results from the Kruskal–Wallis test comparing the mean scores of the 11 ALS–long pass filter combinations within the temperature groups from 220 up to 1100 °C

Temperature	*N* samples	Chi-squared	df	Asymp. sig.
220 °C (10, 20 and 30 min)	16	0.000	10	1.000
250 °C (10, 20 and 30 min)	18	22.842	10	0.011
300 °C (10, 20 and 30 min)	17	6.106	10	0.806
350 °C (10, 20 and 30 min)	16	1.065	10	1.00
400 °C (10, 20 and 30 min)	18	0.598	10	1.00
450 °C (10, 20 and 30 min)	18	0.545	10	1.000
500 °C (10, 20 and 30 min)	14	1.539	10	0.999
600 °C (10, 20 and 30 min)	14	34.017	10	0.000
700 °C (10, 20 and 30 min)	13	12.060	10	0.281
800 °C (10, 20, 30 and 120 min)	14	11.310	10	0.334
900 °C (10, 20, 30 and 150 min)	20	31.013	10	0.001
1000 °C (30 and 150 min)	4	21.775	10	0.016
1100 °C (10 and 120 min)	4	1.422	10	0.999

**Table 3:** Mean ranks, based on the intensity for the 11 ALS–long pass filter combination, for the temperature–duration groups that proved to have a significant difference based on the Kruskal–Wallis test (Table 2)

	Temperature group							
	250 °C for 10, 20 and 30 min		600 °C for 10, 20, and 30 min		900 °C for 10, 20, 30 and 150 min		1000 °C for 30 and 150 min	
ALS + long pass filter	*N*	Mean rank	*N*	Mean rank	*N*	Mean rank	*N*	Mean rank
350 to 380 nm	17	79.29	14	65.54	20	121.00	4	30.50
400 to 430 + 435 ± 6 nm	17	90.03	14	91.71	20	105.75	4	21.50
400 to 430 + 476 ± 6 nm	17	78.82	14	91.71	20	108.83	4	21.50
400 to 430 + 529 ± 6 nm	17	86.06	14	86.18	20	95.35	4	21.50
400 to 430 + 571 ± 6 nm	17	90.79	14	51.71	20	58.63	4	17.75
420 to 470 + 476 ± 6 nm	17	101.50	14	117.79	20	157.70	4	36.75
420 to 470 + 529 ± 6 nm	17	101.50	14	77.00	20	131.43	4	21.50
420 to 470 + 572 ± 6 nm	17	101.50	14	63.21	20	102.15	4	14.00
445 to 510 + 529 ± 6 nm	17	101.50	14	89.46	20	125.88	4	37.00
445 to 510 + 571 ± 6 nm	17	101.50	14	63.21	20	112.55	4	17.75
480 to 560 + 571 ± 6 nm	17	101.50	14	54.96	20	86.25	4	7.75

### Analysis of the remains collected after a modern cremation

**Fig. 8:**
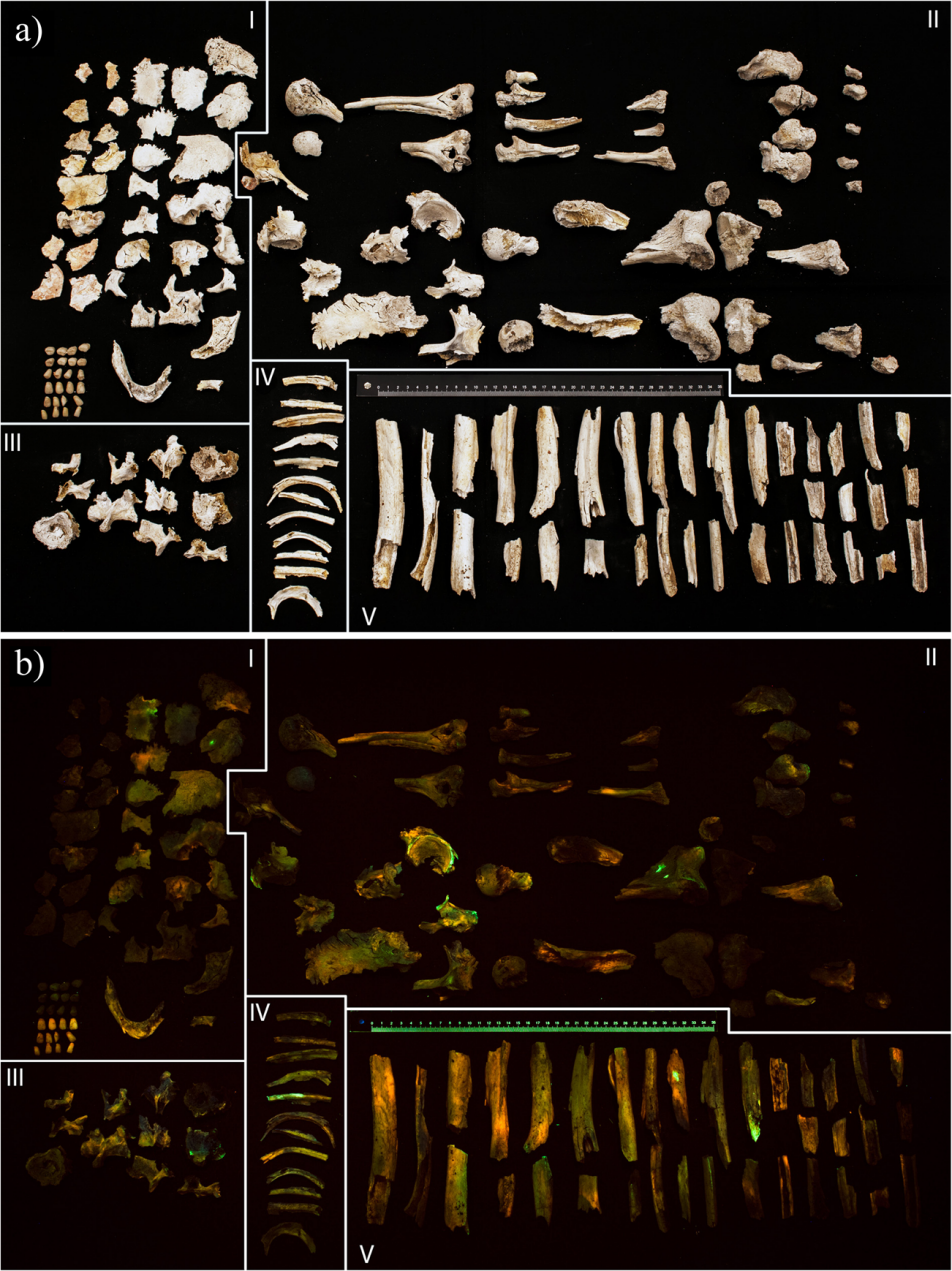
Salvaged cremated remains of one of the cadavers (77-year-old female), cremated at 1000 °C for 2.5 h. Remains are categorized as follows: cranial bones and teeth (*I*), epiphyses and irregluar bones (*II*), vertebrae (*III*), ribs (*IV*) and diaphyseal fragments (*V*). **a** Visualized under white light. **b** Illuminated with a bandwidth of 420 to 470 nm and photographed through a 476 ± 6-nm long pass filter. The remains display a heterogeneously distributed type of luminescence that ranges between present and absent

**Fig. 9:**
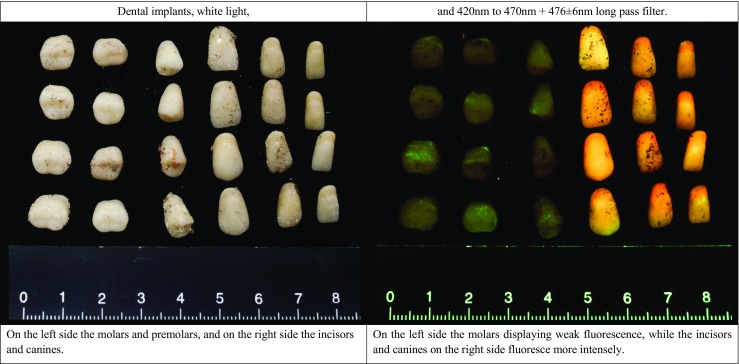
Close-up of the recollected dental implants of the 77-year-old female

The cremated remains of four individuals showed a heterogeneous distribution of luminescence (Fig. 8). The remains of the cadaver that was frozen for 78 days prior to cremation did not deviate from the non-frozen cadavers. Luminescence was observable with every ALS–long pass filter combination used in the experiments, but the blue ALS with yellow long pass filter combination resulted in the highest intensity of luminescence. The scores ranged between 3 (present) and 1 (absent), and different colours of luminescence were observed. The inner table of the cranium displayed a less intense luminescence than the outer table; this was observed in all four cases. Interestingly, the recollected dental implants also luminesced (Fig. 9). The molars and premolars luminesced weak, while the canines and incisors luminesced stronger and at a different colour.

## Discussion

The thermal stress that was applied during the heating experiments in an oven, with or without adipose tissue, cannot be directly compared to the thermal stress that skeletal remains are exposed to during, for example, a house fire. However, in a house fire, the skeletal remains do go through similar thermally induced phases, and it is expected that the findings of the experimentally heated samples will reflect those that can be expected in the field.

The observed luminescent characteristics of bone are strongly related to the colour of the bone samples heated in air, when illuminated with white light. The colour of thermally altered bone is related to the destruction of the organic component of the composite matrix at temperatures at and below 400 °C. A longer duration at temperatures below 400 °C led to more carbonization of the organic matrix and a lower observed intensity of luminescence. Depending on the degree of carbonization of the organic matrix, the accumulated carbon might absorb the excitation light, which could explain the decrease in observed intensity. A longer duration at temperatures higher than 450 °C, and up to 800 °C, led to a higher intensity of observed luminescence, due to the combustion of the remnants of the organic compounds. Size of the sample, and thus amount of organic material, might explain why temperature 600 °C deviated from this trend. A longer duration at 900 °C led to a reduction in intensity of observed luminescence, suggesting that the chemical composition of the inorganic compound is being altered, an explanation that is further substantiated with a change in the colour of the luminescence which was noted from 800 °C for 30 min and higher temperatures. Samples heated in adipose tissue, which restricted the amount of available oxygen, retained their luminescent characteristic up to a higher temperature and for a longer duration. The latter substantiates the hypothesis that the presence of air, next to temperature and duration, is also a major factor for changes of the organic matrix. It cannot be excluded that thermal decomposition of the organic matrix in combination with oxidation of lipids can lead to changes in luminescence and observed intensity [29].

Changes in luminescence can be explained by changes in the chemical composition of the bone material. Since changes, such as the spectral shift and the fading luminescence, occur after the organic matrix has been thermally decomposed, it appears that these changes must take place within the inorganic matrix. The formation of new mineral phases, like CaO and β-TCP, is dependent on the Ca/P molar ratio (>1.67) [30]. The chemical conversion of CHA to β-TCP was suggested to occur in (non-human) bone heated at temperatures from 600 °C and higher, by Civjan et al. and Bonucci et al. [31, 32]. These findings were not confirmed by X-ray diffraction (XRD) experiments carried out by Rogers et al., who heated human cortical bone sections in air in the range of 200 to 1200 °C; this study only reported the presence of CHA and CaO [33]. A later XRD study by Beckett et al., who also heated human cortical bone sections, showed that β-TCP was not present in samples heated to 600 °C but that a substantial fraction was found in samples heated to 1400 °C [34]. Therefore, it is expected that a chemical conversion from CHA to β-TCP in bone is not the underlying cause for the shift in colour of luminescence of the thermally altered bone samples from 800 °C and higher, especially because samples heated to 1100 °C for a relative long duration do not luminesce at all.

Another explanation for the changes in luminescence might be the re-crystallization of the inorganic matrix due to thermal stress. Herrmann described a change of the lamellar structure of cortical bone in to a homogenous texture in completely cremated bone for temperatures higher than 800 °C [35]. Later, Holden et al. observed the formation of new crystals with a hexagonal morphology at temperatures between 800 and 1400 °C and a duration of 2 h with fresh human cortical bone. These hexagonal crystals increased in size with increasing temperature, and between 1000 and 1400 °C, these crystals started to fuse [36]. Piga et al. showed by means of XRD that the CHA crystals, of heated human dry bone, start to grow at a temperature of 700 °C, and it became most evident in the range of 750 to 850 °C and a duration of less than 1 h [37]. Figueiredo et al. have confirmed the increase in crystal size of CHA in heat-treated human bone and thermal decomposition of carbonate, resulting in an increasing purity of the inorganic matrix, for longer durations [38]. But, diagenesis can also lead to an increase in crystallite size, thereby mimicking the effect of thermal stress [39, 40]. Further, but not limited to, Ramstahler et al., Hoke et al., and Swaraldahab et al. have shown that the fluorescence of both non-human and human bone changes in colour from blue to yellow (varying shades have been observed) and decreases in intensity to, in some cases, a complete absence when excited with UV, as the postmortem interval increases [26, 41, 42]. Thus, it is expected that an increase in crystallite size might explain the temperature-dependent and duration-dependent decrease in observed intensity of luminescence at temperatures higher than 800 °C (Fig. 6). The colour shift observed at temperatures of 800 °C and above has not been associated with changes in luminescence caused by a prolonged postmortem interval. Therefore, the cause for the observed shift in luminescence has yet to be identified, since it is not easily explained by the chemical conversions or the changes in crystallite size. The cremated human skeletal remains that Mavin investigated actually originated from an archaeological excavation, which might explain the discrepancy between his negative findings and the results of the present study which shows that cremated human bone does luminesce in most cases (*personal communication 14 Sept. 2015)*.

The remains from the crematory exhibited diversity in both intensity and colour of luminescence. This finding sheds doubt on the conclusion of Warren et al. regarding the relation between the luminescence and the “age” of the cremated remains and the used cremation oven [16]. A rather large difference in intensity and colour can already be observed among the remains of one cremated cadaver from one oven. The retained luminescence of the dental implants, after exposure to heat, increases the chance of their retrieval when an ALS is used in the investigation, and dental implants greatly improve the chance for identification of fragmentary remains. However, the composition of dental implants is brand and type specific, as is, therefore, the thermal alteration of the dental implants [43, 44].

Since cremated human bone does luminesce, in the majority of the investigated temperature ranges, it should be possible to distinguish bone from non-luminescent materials in difficult contexts. Bone carbonizes when heated within the range of 350 to 450 °C, both in air and in adipose tissue; under white light, these samples appear brown to black. However, when illuminated with an ALS, the samples heated in adipose tissue showed a higher intensity of luminescence than samples heated in air. A spectral shift in emission bandwidth occurred around 800 °C. This shift is specific for periosteal bone and is not observed for transverse sections. As samples heated to temperatures above 700 °C turn chalky white, it can be difficult, if not impossible, to distinguish temperature above 700 °C. However, using an ALS, the presence of a spectral shift shows that a sample has been heated to at least 800 °C for 30 min. Based on the current results, it is therefore advisable to use an ALS to retrieve and investigate human remains that have been exposed to heat.

## Conclusion

The actual spectral bandwidths of the used ALS, which exceeded the cut-off wavelength of some of the used long pass filters, hampered differentiation between luminescence with a narrow emission bandwidth and reflectance, or a combination of both. Nonetheless, the observations were quantifiable with a high level of agreement between observers. The luminescent characteristics of bone were observable for almost all temperature–duration combinations, except for samples heated in the range of 350 °C for 20 min to 400 °C for 20 min and 1100 °C for 210 min. A spectral shift in luminescence was observed for samples heated to 800 °C for 30 min and higher. While the underlying cause for this observed shift is still unknown, the temperature, duration and the amount of available oxygen do have a significant effect on the observed intensity of luminescence. Based on statistical analysis, the blue ALS (420 to 470 nm) with a yellow long pass filter (476 ± 6 nm) leads to the best results over the full range of temperatures and durations that were investigated. Samples recovered from the crematory showed a heterogeneous range of the investigated luminescence characteristics (both in colour and intensity). In conclusion, an ALS can aid the gathering of information on the perimortem and postmortem events and in the recovery of cremated human remains. To enable more detailed conclusions on subtler temperature-related and duration-related changes and to further investigate the temperature-dependent and duration-dependent intensity and emission bandwidth, the scoring index used in this study should be extended to spectroscopic analysis.

## Electronic supplementary material

ESM 1 (DOCX 259 kb)
