# Supplementary material for: Luminescence of thermally altered human skeletal remains
Source: Int J Legal Med. 2017 Feb 23;131(4):1165–77. doi: 10.1007/s00414-017-1546-1 (PMC5491595; doi:10.1007/s00414-017-1546-1)
Supplement: Supplementary file 1 — (DOCX 259 kb) [file 414_2017_1546_MOESM1_ESM.docx]

**Electronic Supplement Material**

**Rise from the ashes: Luminescence of thermally altered human skeletal remains**

*International Journal of Legal Medicine*

Tristan Krap ^1,2,3^, Kevin Nota ^2^, Leah S. Wilk ^4,5^, Franklin R.W. van de Goot ^6^, Jan M. Ruijter ^1^, Wilma Duijst ^3,7^, Roelof-Jan Oostra ^1^

*1: Department of Anatomy, Embryology & Physiology, Academic Medical Centre, Amsterdam, The Netherlands. *2: Department of Life Sciences & Technology – Biotechnology – Forensic Science, Van Hall Larenstein, University of Applied Sciences, Leeuwarden, The Netherlands. *3: Ars Cogniscendi Centre for Legal and Forensic medicine, Wezep, The Netherlands. *4: Department of Biomedical Engineering and Physics, Academic Medical Centre, Amsterdam, The Netherlands. *5: Forensic Technical Solutions B.V., Amsterdam, The Netherlands. *6: Centre for Forensic Pathology, Baarn, The Netherlands. *7: Netherlands Forensic Institute (NFI), The Hague, Netherlands.

Corresponding author:

Name: Tristan Krap

Email: [T.Krap@amc.nl](mailto:T.Krap@amc.nl)

**A: Details concerning sample size**

Table 1 shows the heating scheme and corresponding sample sizes for the transverse cross sections, and table 2 shows the heating scheme and corresponding sample sizes for the thick diaphyseal sections and epiphyseal ends.

Table 1. Heating scheme for the transverse cross-sections, including the surrounding media.

| **Temperature:** | **Surrounding medium:** | **Duration:** | **N (sample):** |
| --- | --- | --- | --- |
| Unheated | - | - | 5 |
| 100°C | Air  Adipose tissue | 10/20/30 minutes  10/20/30 minutes | 2/2/4  2/2/4 |
| 160°C | Air  Adipose tissue | 10/20/30 minutes  10/20/30 minutes | 2/2/2  2/2/2 |
| 180°C | Air  Adipose tissue | 10/20/30 minutes  10/20/30 minutes | 2/2/4  2/2/4 |
| 200°C | Air  Adipose tissue | 10/20/30 minutes  10/20/30 minutes | 2/2/4  2/2/4 |
| 220°C | Air  Adipose tissue | 10/20/30 minutes  10/20/30 minutes | 2/2/4  2/2/4 |
| 250°C | Air  Adipose tissue | 10/20/30 minutes  10/20/30 minutes | 2/2/4  2/2/4 |
| 300°C | Air  Adipose tissue | 10/20/30 minutes  10/20/30 minutes | 2/2/4  2/2/4 |
| 350°C | Air  Adipose tissue | 10/20/30 minutes  10/20/30 minutes | 2/2/4  2/2/4 |
| 400°C | Air  Adipose tissue | 10/20/30 minutes  10/20/30 minutes | 2/4/2  2/4/2 |
| 450°C | Air  Adipose tissue | 10/20/30 minutes 10/20/30 minutes | 2/2/4  2/2/4 |
| 500°C | Air | 10/20/30 minutes | 4/4/4 |
| 600°C | Air | 10/20/30 minutes | 4/4/4 |
| 700°C | Air | 10/20/30 minutes | 4/4/4 |
| 800°C | Air | 10/20/30 minutes | 4/4/4 |
| 900°C | Air | 10/20/30 minutes | 4/4/4 |
| Total: | | | 229 |

Table 2. Heating scheme for the thick diaphyseal sections and epiphyseal ends in medium air.

| **Temperature:** | **Surrounding medium:** | **Duration:** | **N (sample):** |
| --- | --- | --- | --- |
| Unheated | - | - | 1 (diaphysis) |
| 250°C | Air | 30 minutes | 1 (diaphysis) |
| 300°C | Air | 30 minutes | 1 (diaphysis) |
| 400°C | Air | 20 minutes | 2 (1 (diaphysis)/1 epiphysis) |
| 450°C | Air | 30 minutes | 2 (diaphysis) |
| 500°C | Air | 30 minutes | 2 (diaphysis) |
| 600°C | Air | 30 minutes | 2 (diaphysis) |
| 700°C | Air | 30 minutes | 1 (diaphysis) |
| 800°C | Air | 30 minutes  120 minutes | 1 (epiphysis)  1 (epiphysis) |
| 900°C | Air | 10 minutes  20 minutes  30 minutes  150 minutes | 2 (diaphysis)  2 (epiphysis)  2 (epiphysis)  2 (epiphysis) |
| 1000°C | Air | 30 minutes  150 minutes | 2 (diaphysis)  2 (epiphysis) |
| 1100°C | Air | 10 minutes  120 minutes  210 minutes | 2 (epiphysis)  2 (epiphysis)  1 (epiphysis) |
| Total: | | | 31 |

**B: Spectral output analysis of the alternate light sources.**

Figure 1.A to 1.E demonstrate that the spectral output of the ALS exceeds the respective nominal cut-off wavelengths provided by the manufacturer. The specific cut-off wavelengths are indicated in the figures by a red line. Figures 1.A to 1.D further show a small peak in the nominal spectral range of the respective ALS. This peak corresponds to the nominal ALS output that is not entirely filtered out by the used long-pass filter due to the ALS’s high output intensity.

Figure 1.

| A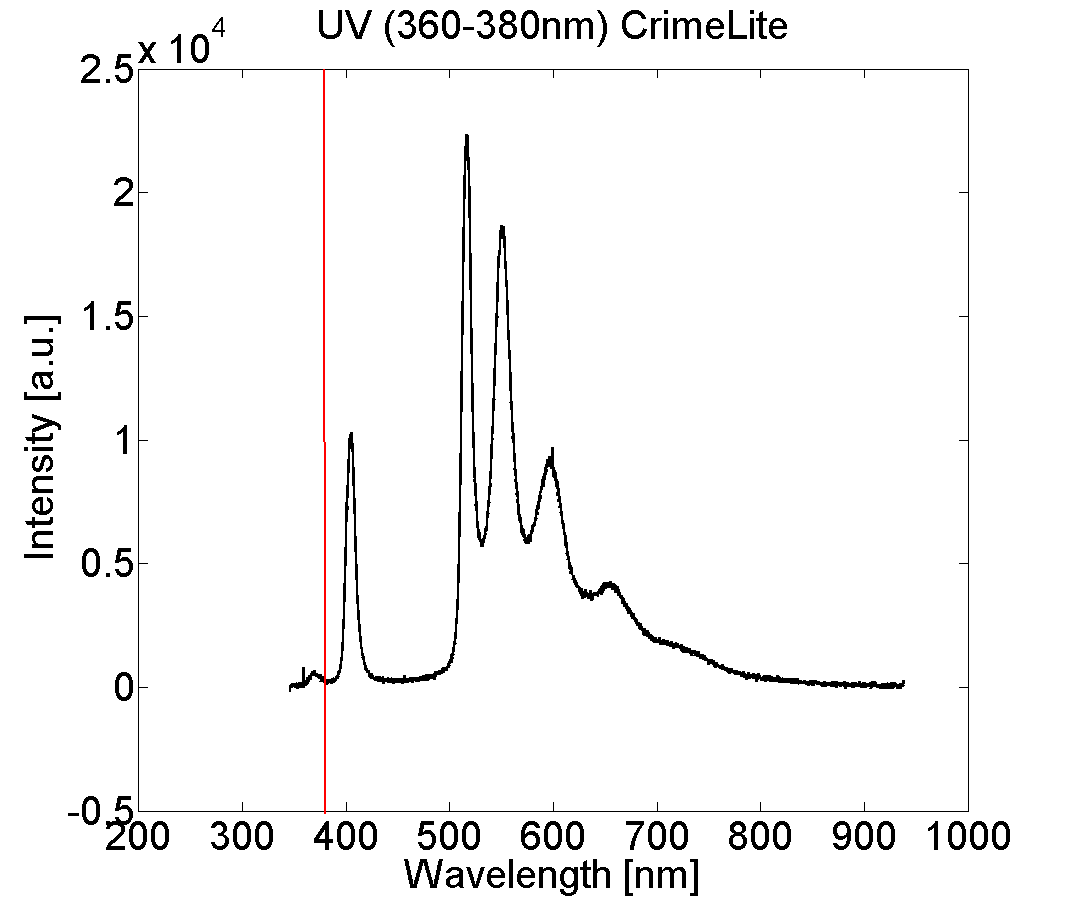 | B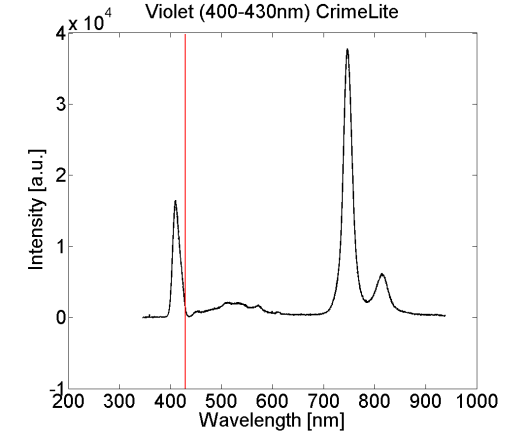 | C**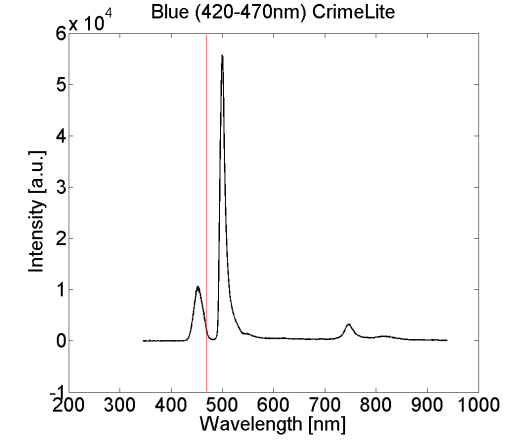** |
| --- | --- | --- |
| D **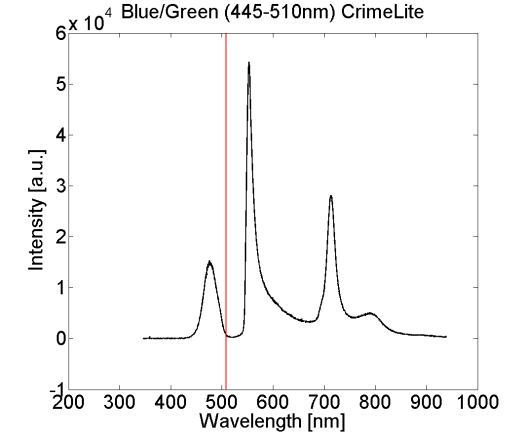** | E*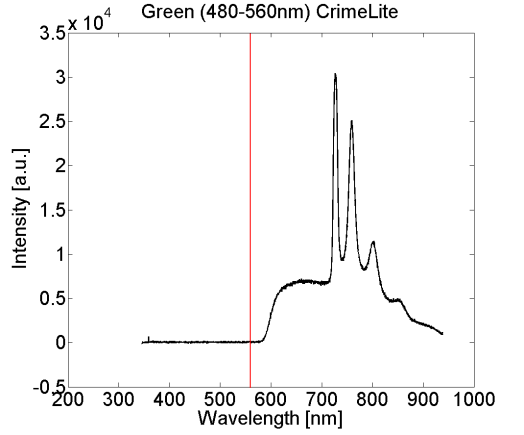* |  |

**C: Intra- and inter-observer error.**

This section provides details concerning the obtained Kappa tests for the scores of the two observers. Table 3 shows the intra-observer error for the two observers and table 4 shows the inter-observer error between both observers for both scores.

Table 3. Intra-observer error, Kappa agreement value based on the first versus the second score of the observers.

|  | | Symmetric Measures: First versus second score TK | | | | Symmetric Measures: First versus second score KN | | | |
| --- | --- | --- | --- | --- | --- | --- | --- | --- | --- |
|  |  |  |  |  |  |  |  |  |  |
|  |  | Value | Asymp. Std. Error^a^ | Approx. T^b^ | Approx. Sig. | Value | Asymp. Std. Error^a^ | Approx. T^b^ | Approx. Sig. |
| Measure of Agreement | Kappa | 0.961 | 0.004 | 82.233 | 0.000 | 0.949 | 0.005 | 82.326 | 0.000 |
| N of Valid Cases | | 2860 |  |  |  | 2860 |  |  |  |
| a. Not assuming the null hypothesis. | | | | | | | | | |
| b. Using the asymptotic standard error assuming the null hypothesis. | | | | | | | | | |

Table 4. Inter-observer error, Kappa agreement value based on the scores between observers.

|  | | Symmetric Measures: First score TK versus first score KN | | | | | Symmetric Measures: First score TK versus second score KN | | | |
| --- | --- | --- | --- | --- | --- | --- | --- | --- | --- | --- |
|  |  |  |  |  |  |  |  |  |  |  |
|  |  | Value | Asymp. Std. Error^a^ | | Approx. T^b^ | Approx. Sig. | Value | Asymp. Std. Error^a^ | Approx. T^b^ | Approx. Sig. |
| Measure of Agreement | Kappa | 0.870 | 0.008 | | 75.177 | 0.000 | 0.870 | 0.008 | 75.019 | 0.000 |
| N of Valid Cases | | 2860 |  | |  |  | 2860 |  |  |  |
|  | | Symmetric Measures: Second score TK versus first score KN | | | | | Symmetric Measures: Second score TK versus second score KN | | | |
|  |  |  |  |  |  |  |  |  |  |  |
|  |  | Value | Asymp. Std. Error^a^ | Approx. T^b^ | | Approx. Sig. | Value | Asymp. Std. Error^a^ | Approx. T^b^ | Approx. Sig. |
| Measure of Agreement | Kappa | 0.875 | 0.007 | 75.437 | | 0.000 | 0.892 | 0.007 | 76.810 | 0.000 |
| N of Valid Cases | | 2860 |  |  | |  | 2860 |  |  |  |
| a. Not assuming the null hypothesis. | | | | | | | | | | |
| b. Using the asymptotic standard error assuming the null hypothesis. | | | | | | | | | | |
